# Genome-Wide Association Study Reveals Genomic Regions Associated with Fusarium Wilt Resistance in Common Bean

**DOI:** 10.3390/genes12050765

**Published:** 2021-05-18

**Authors:** Jean Fausto de Carvalho Paulino, Caléo Panhoca de Almeida, César Júnior Bueno, Qijian Song, Roberto Fritsche-Neto, Sérgio Augusto Morais Carbonell, Alisson Fernando Chiorato, Luciana Lasry Benchimol-Reis

**Affiliations:** 1 Centro de Recursos Genéticos Vegetais, Instituto Agronômico, Campinas 13075-630, SP, Brazil; jeanbiotec@gmail.com (J.F.d.C.P.); caleoalmeida@hotmail.com (C.P.d.A.); 2 Centro Avançado de Pesquisa em Proteção de Plantas e Saúde Animal, Instituto Biológico, Campinas 13101-680, SP, Brazil; cesar.bueno@sp.gov.br; 3 Soybean Genomics and Improvement Laboratory, US Department of Agriculture, Agricultural Research Service (USDA-ARS), Beltsville, MD 20705, USA; qijian.song@usda.gov; 4 Department of Genetics, ‘Luiz de Queiroz’ Agriculture College, University of Sao Paulo, Piracicaba 13418-900, SP, Brazil; roberto.neto@usp.br; 5 Centro de Grãos e Fibras, Instituto Agronômico, Campinas 13075-630, SP, Brazil; sergio.carbonell@sp.gov.br (S.A.M.C.); alisson.chiorato@sp.gov.br (A.F.C.)

**Keywords:** *Phaseolus vulgaris* L., *Fusarium oxysporum* f. sp. *phaseoli*, SNP markers, disease resistance, molecular breeding

## Abstract

Fusarium wilt (*Fusarium oxysporum* f. sp. *phaseoli*, *Fop*) is one of the main fungal soil diseases in common bean. The aim of the present study was to identify genomic regions associated with *Fop* resistance through genome-wide association studies (GWAS) in a Mesoamerican Diversity Panel (MDP) and to identify potential common bean sources of *Fop*’s resistance. The MDP was genotyped with BARCBean6K_3BeadChip and evaluated for *Fop* resistance with two different monosporic strains using the root-dip method. Disease severity rating (DSR) and the area under the disease progress curve (AUDPC), at 21 days after inoculation (DAI), were used for GWAS performed with FarmCPU model. The *p*-value of each SNP was determined by resampling method and Bonferroni test. For UFV01 strain, two significant single nucleotide polymorphisms (SNPs) were mapped on the Pv05 and Pv11 for AUDPC, and the same SNP (ss715648096) on Pv11 was associated with AUDPC and DSR. Another SNP, mapped on Pv03, showed significance for DSR. Regarding IAC18001 strain, significant SNPs on Pv03, Pv04, Pv05, Pv07 and on Pv01, Pv05, and Pv10 were observed. Putative candidate genes related to nucleotide-binding sites and carboxy-terminal leucine-rich repeats were identified. The markers may be important future tools for genomic selection to *Fop* disease resistance in beans.

## 1. Introduction

Common bean (*Phaseolus vulgaris* L.) originated in Mexico 4 to 6 million years ago [1] and was independently domesticated in Mesoamerica and the Andes 8000 years ago, constituting two main known gene pools [2]. Beans refers to legumes of the genus *Phaseolus*, family Fabaceae, subfamily Papilionoideae, tribe Phaseoleae, and subtribe Phaseolinae [3]. Common bean (*Phaseolus vulgaris* L.) is a diploid (2n = 2× = 22), annual, predominantly self-pollinating species and one of the most important pulses worldwide [4,5].

Based on nucleotide sequences of chloroplasts, patterns of phaseolins, and genetic signatures in domesticated and wild accessions, it appears that the greatest genetic variation occurs among genotypes of the Mesoamerican gene pool, the most preferred type of bean for consumption in Brazil [6,7,8,9]. According to the FAO (Food and Agriculture Organization of the United Nations), global production of dry bean in 2018 was approximately 31.5 million tons. Brazil is considered the third largest producer in the world, with production of approximately 3.1 million tons [10]. The favorable edaphic and climatic conditions for growing common bean in Brazil allow wide distribution in every Brazilian state, with different harvest seasons, which is key for an annual supply [11].

An increase in planted area, especially under irrigation, combined with multiple crop seasons, has created conditions for high incidence of soil diseases, which are among the main causes of low crop yield and considerable losses [12]. One of the main fungal diseases, Fusarium wilt, is a severe vascular disease in common bean whose causal agent is *Fusarium oxysporum* Schlecht. f. sp. *phaseoli* Kendrick & Snyder (*Fop*) [13,14]. The infection process begins in the roots, colonizes the xylem, and causes leaf wilt, vascular discoloration, chlorosis, dwarfism, and premature plant death [15,16].

Therefore, it is necessary to identify potential bean sources of resistance to effectively control the pathogen. The development of resistant cultivars is a promising alternative for control of this disease as resistant cultivars are easily adopted by producers and do not cause environmental risks [17,18]. Pathogenicity testing through inoculation methods can be used to characterize the degree of pathogenicity of *Fop* strains [19]. Pathogenicity testing also provides an alternative for assessing the diversity of physiological races of the pathogen, the main cause of breakdown in genetic resistance to *Fop* in bean cultivars [20,21]. Currently, several definitions describe the complexity of genetic resistance of bean to *Fop*; some studies report it to be monogenic [22,23], some as oligogenic [24,25,26], and another as polygenic [17].

In common bean, only a limited number of studies have been conducted with the goal of clarifying the comprehension of the molecular mechanisms and pathways involved in bean response to *Fop*’s infection [27]. Recent results demonstrate by Chen et al. [27] using whole transcriptome and metabolome of common bean infected by *Fop* shows the response to *Fop* uses different and effective defense pathways comprising of a complex resistance network of structural, signaling, hormonal and chemical responses.

An alternative for understanding genetic control of bean resistance to *Fop*, how resistance loci are distributed in the bean genome, and the intensity of their effects is to study them indirectly, through their association with genetic markers [28,29]. Among the preferred genetic markers, single nucleotide polymorphisms (SNPs) are noteworthy, since these markers can be integrated with the QTN (Quantitative Trait Nucleotide) responsible for phenotypic variation in the trait of interest [30].

The GWAS using natural populations have higher mapping resolution than linkage mapping and greater cost-effectiveness [31]. In GWAS, the recombination events accumulated over innumerable generations reduce linkage disequilibrium (LD), allowing more precise estimates of the location of genes of interest to be obtained [32]. In recent years, GWAS has been widely used to investigate the genetic architecture of complex characteristics in model plants such as Arabidopsis thaliana [33], soybean [34,35], and common bean [36,37].

In the current study, a total of 2001 high-quality single-nucleotide polymorphisms (SNPs) distributed over the 11 bean chromosomes were genotyped using SNP Assay technology (Illumina BARCBean6K_3 BeadChip) [38]. The BARCBean6K_3 BeadChip has successfully contributed to the study of several traits in beans [39,40,41,42,43,44]. This technology has also been used in the identification of genomic regions associated with disease resistance, such as common mosaic virus [45], anthracnose [41], root rot [46], rust [42], angular leaf spot [47], and to fusarium wilt [25].

The aim of the present study was to identify new genomic regions associated with *Fop* resistance in a Mesoamerican Diversity Panel (MDP) and, taking into account the phenotypical evaluations, identify potential genotypes of common bean as sources for *Fop*’s resistance.

## 2. Materials and Methods

### 2.1. Plant Material

A total of 205 common bean genotypes of Mesoamerican origin with characteristics of agronomic interest mainly concerning disease resistance and the technological quality of the bean grain were selected from the germplasm bank (BAG) of the Agronomic Institute (IAC, Campinas, SP, Brazil) to represent the MDP. These accessions were chosen taking into account the genetic characterization of Mesoamerican accessions that had been carried out in advance [37,48].

In Brazil, there is a greater preference for Mesoamerican beans [49]. The MDP includes accessions that were released by common bean breeding programs form different institutions in Brazil, both private and governmental. Altogether, 131 genotypes belonging to the Carioca commercial class, validated in advance by GWAS [37]. In addition, 30 genotypes from the Black commercial class, and 44 genotypes from the Special class including ‘Pinto’, ‘Cream’, ‘Mottled’, ‘Mulatinho’, ‘Rose’, ‘Red’ ‘Light Pink’, ‘Brown’, ‘Light brown’, ‘Yellow’, ‘Red’ (Table S1).

### 2.2. DNA Extraction, Genotyping and SNP Calling

The total genomic DNA of each sample from the MDP was extracted from young leaves using the CTAB protocol [50]. The quality of the DNA was confirmed by electrophoresis in 1% agarose and quantified by the Qubit fluorimeter (Thermo Fisher, Waltham, MA, USA). All samples were diluted to a concentration of 50 ng·μL^−1^. Genotyping was performed using the BeadChip BARCBean6K_3 technology with 5398 SNPs [38]. The BARCBean6K_3 was developed based on the first common bean genome (i.e., *Phaseolus vulgaris* v1), and subsequently, the flanking sequences of each SNPs were blasted (e.g., BLASTN) against the most current reference genome, *Phaseolus vulgaris* v2.1 [51], and the position of each SNP was obtained. The SNP calling and genotypic data obtained were analyzed for quality using the Genome Studio 2.0 software (Illumina, San Diego, CA, USA).

The genotype matrix was converted into HapMap format, with the reference allele represented by “A”, the alternative allele by “G”, the heterozygous allele by “R”, and the missing data by “N” using the TASSEL 5.0 software [52]. For the quality control, SNPs with MAF (Minor Allele Frequency) smaller than 0.05, heterozygosity, and missing data greater than 0.10 were removed. Finally, markers not positioned in the genome were also removed, and “N” loci were imputed using the Beagle 5.0 software [53]. After the quality control filters, 2001 high-quality SNPs were selected for association mapping.

### 2.3. Inoculation and Evaluation of Fop Strains in Common Beans

The 205 MDP genotypes were evaluated for *Fop* resistance under greenhouse conditions in a randomized complete block design. Each genotype was replicated three times. A replicate was represented by one plastic pot with dimensions of 11 × 8 × 9 cm^3^ containing two plants of a single genotype, for a total of six repetitions evaluated for each genotype. The genotypes were planted in 128-cell trays containing sterile vermiculite.

Among the genotypes, IAC Milênio was used as a *Fop*-resistant check cultivar and BRS Estilo as a susceptible check cultivar [19]. Two strains of *Fop* were used. The first (UFV01 strain) was collected from the Meia Noite cultivar in Coimbra, Minas Gerais, Brazil [14] and the second (IAC18001 strain) was obtained from the A211 cultivar in Campinas, São Paulo, Brazil. Both strains were purified through a single spore from cultures previously confirmed as new races of *Fop* [54,55].

The inocula were produced on PDA (potato-dextrose-agar) medium incubated for 10 days in a growth chamber at 24 ± 1 °C with a 12-h photoperiod. The spore suspension was prepared one hour before inoculation at the concentration of 1 × 10^6^ conidia mL^−1^, including macro and microconidia [56].

Ten days after sowing, the roots of each genotype were washed using distilled water and a third of the length was cut using a sterile scissor (the root-dip method, Paulino et al. [19]. The roots were immediately immersed in a Falcon^®^ tube containing ten mL of the spore suspension for five minutes. The roots of the control cultivars were immersed in autoclaved water for the same time. The plants were transplanted to a plastic pot containing the substrate Biomix^®^, followed by the addition of 10 mL of inoculum, and kept in a greenhouse until the time of evaluation. The plants were irrigated daily, and each pot was fertilized with 0.3 g urea as N source at ten DAI.

**Figure 1:**
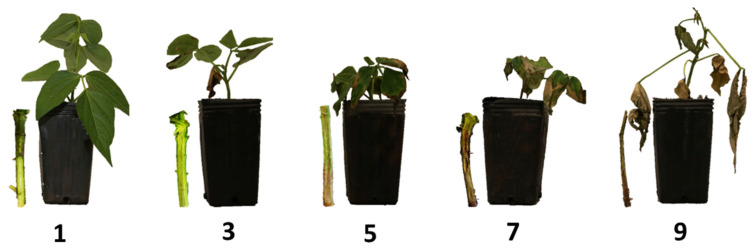
The DSR was measured according to an adapted scale [56], with values ranging from 1 to 9: score 1 = absence of symptoms and discoloration in the hypocotyl; score 3 = chlorosis, wilt, and restricted necrosis of the first leaves of the plant, with slight discoloration in the hypocotyl; score 5 = chlorosis, wilt, and necrosis in the leaves below the pointer and intermediate discoloration in the hypocotyl; score 7 = severe symptoms of generalized wilting throughout the plant, and dwarfism and severe discoloration in the hypocotyl; and score 9 = dead plant.

After the appearance of the first symptoms, at 14 DAI, evaluations were performed at 15, 18, and 21 DAI, with equal intervals between evaluations. The DSR was measured according to an adapted scale [56], with values ranging from 1 to 9: score 1 = absence of symptoms and discoloration in the hypocotyl; score 3 = chlorosis, wilt, and restricted necrosis of the first leaves of the plant, with slight discoloration in the hypocotyl; score 5 = chlorosis, wilt, and necrosis in the leaves below the pointer and intermediate discoloration in the hypocotyl; score 7 = severe symptoms of generalized wilting throughout the plant, and dwarfism and severe discoloration in the hypocotyl; and score 9 = dead plant (Figure 1). The genotypes were classified according to resistance to the strain using the following criteria: resistant—scores from 1 and 3; moderately resistant—scores from 3.1 to 6.0; and susceptible—scores from 6.1 to 9.0.

The disease severity data for all evaluations for each genotype were used to calculate the *AUDPC* by Shaner and Finney [57] according to the formula:

$$AUDPC=\overset{n}{\underset{i=1}{\sum }}\left[\left(\frac{Y_{i+1}+Y_{i}}{2}\right)\;\left(T_{i+1}+T_{i}\right)\right]$$

where

*Y_i_* = severity of *Fop* at the *i*th observation,

*T_i_* = time (DAI) at the *i*th observation and

*n* = total number of evaluations.

### 2.4. Statistical Analysis and Prediction of Genotipic Values

The DSR and AUDPC were compared using Pearson correlation at 21 DAI. The linear mixed model applied was:

Trait (DSR, AUDPC)=accession+block+error

The assumptions of normal errors and homogeneous error variance were checked. In a first step, we carried out analysis of deviance (ANADEV) by the likelihood ratio test (LRT) method. The linear mixed model was used, and in a first step, the broad-sense heritability and accession effect vector that was considered as random. In a second step, the accession effect vector was considered as fixed, and the phenotypic matrix was given by the genotypic values estimated by the Restricted Maximum Likelihood/Best Linear Unbiased Estimator-REML/BLUE of the Be-Breeder package [58]. The genotypic values for each accession and trait were used as input phenotypic data in association mapping analysis.

### 2.5. Genome-Wide Association Studies

A fixed and random model Circulating Probability Unification—FarmCPU—was used in GWAS [59]. The package explores the MLMM (multi-locus mixed-model) and performs analysis in two interactive steps: a fixed-effect model (FEM) is applied first, followed by a random-effect model (REM), so that both are repeated interactively until no significant SNP is detected. To avoid type I errors (i.e., false positives), the structuring matrix was tested using the Bayesian Information Criterion (BIC) test according to Schwarz [60] for a regular mixed linear model available in GAPIT 2.0 [61] with the first five components of the PCA. The population structure of MDP (structure results derived from PCA and BIC test) and the relatedness to Kinship (heatmap) [62] were included in the GWAS model.

The limit of the *p*-value of each SNP was determined by the resampling method using the FarmCPU P Threshold function. Each trait was exchanged 1000 times to break the relationship with the genotypes, and then the random association between all SNPs with the phenotype was estimated. The minimum *p*-value was recorded based on all SNPs for the 1000 repetitions, and then the 95% quantile of the entire minimum *p*-value was defined as the limit *p*-value [63]. The Bonferroni test [64] was also used as a threshold for the output in the Manhattan plot, to observe the dispersion of associations between SNP markers and the trait of interest.

### 2.6. Candidate Gene Identification

The significant SNPs were tested with a confidence interval of each SNP for size given by the size of the haplotype blocks in LD (i.e., using r^2^ ≥ 0.2), and the LD was estimated using squared allele-frequency correlation intrachromosomal pairs, through the Gaston package, available in R [65]. The LD decay curves for all chromosomes accessed from MDP was explained using the nonlinear model proposed by Hill and Weir [66], as described by Diniz et al. [48]. The common bean genome sequences were investigated using the BlastN analyses against the reference genome (*Phaseolus vulgaris* v 2.1; Schmutz et al. [51], using Jbrowse on Phytozome [67].

## 3. Results

### 3.1. Evaluation of Fusarium Wilt Severity in Common Beans

Common bean genotypes showed differential interaction when evaluated for resistance to *Fop* of both strains. At 21 DAI, genotypes that showed resistance against strains exhibited symptoms such as wilt, restricted necrosis of the first leaves, and a slight discoloration in the hypocotyl of the plant. However, susceptible genotypes showed wilt, necrosis, and severe discoloration in the hypocotyl, with generalized wilting throughout the plant, dwarfism, and consequently death.

The great variability of the MDP was confirmed by high significance (*p* < 0.01) of ANADEV for all evaluations, validating the use of both phenotypic evaluation for GWAS with respective adjusted means and genotypic data (Table S1. Broad-sense heritability (h^2^) was from 0.48 to 0.63, the lowest value being estimated for AUDPC of the IAC18001 strain (h^2^ = 0.48 ± 0.08) and the highest value for DSR of the UFV01 strain (h^2^ = 0.63 ± 0.09) (Table 1).

The UFV01 strain was more aggressive than IAC18001 in the MDP, with an increase of 17.7% in comparison to the overall mean of the DSR and of 21.8% in comparison to the overall mean of the AUDPC. The results showed that 46.82% (96) of the genotypes evaluated were categorized as resistant, 43.41% (89) as moderately resistant, and 9.77% (20) as susceptible to the UFV01 strain. In relation to the IAC18001 strain, 73% (150) of the genotypes were classified as resistant, 23.90% (49) as moderately resistant, and 3.10% as susceptible (6). Of these, only 36% (75) of the genotypes were resistant to both strains, indicating the possibility of using them as sources of resistance to *Fop* in common bean breeding programs.

The IAC Milênio from the carioca commercial class may be considered an important source of *Fop*’s resistance because it showed resistance to both strains (mean 1.67). It also retains high grain quality, resistance to seed coat darkening, and resistance to *C. lindemuthianum* [68,69]. Previous studies have shown that the cultivar also exhibits correlations between *Fop*’s resistance and the rhizosphere microbiome composition, providing the first line of defense against root infections by soil-borne pathogens [70]. Another important source of resistance to *Fop* is the BRS FC402 cultivar, which is also a carioca cultivar with commercial grain quality and resistance to Fusarium wilt, and which showed a mean of 2.33 for both strains, corroborating the high resistance to *Fop* observed in previous studies [71].

The black seed coat cultivar IPR Uirapuru showed susceptibility to the UFV01 strain, with an average score in the evaluation to both strains of 5.5, corroborating with previous studies. It is a cultivar used as a standard for susceptibility to *Fop* [15]. However, the accessions LEG50600 and RAI 76 derived from CIAT (International Center for Tropical Agriculture, Cali, Colombia) showed good performance for both strains, with an average of 1.45 and 3.0, respectively. Both belong to the black bean commercial class and were previously characterized with root system traits, such as root dry matter, root surface area, and root volume, that showed positive and significant correlations with grain yield under drought [72].

Likewise, some accessions previously characterized as tolerant to water deficit had good performance in resistance to both strains of *Fop*, especially the genotypes BAT-477 [73], SEA-5 [74], SER-16, and IAC Imperador [75], with average scores of 1.33, 1.33, 2.65, and 2.0, respectively. BRS Estilo and A211 showed susceptibility to UFV01 through the root-dip inoculation method, with susceptibility levels like those previously reported [19,55]. This may be explained by the origin of UFV01, which was collected in Coimbra (MG, Brazil) and previously characterized as a highly virulent strain [20,55]. In contrast, IAC18001 was obtained in a naturally infested area at the Santa Elisa Farm of the Agronomic Institute (IAC, Campinas, SP, Brazil), where advanced evaluations and selection of elite bean lines and cultivars are routinely made in competition trials.

### 3.2. Association Mapping for Fusarium Wilt Resistance in the Mesoamerican Diversity Panel

**Figure 2:**
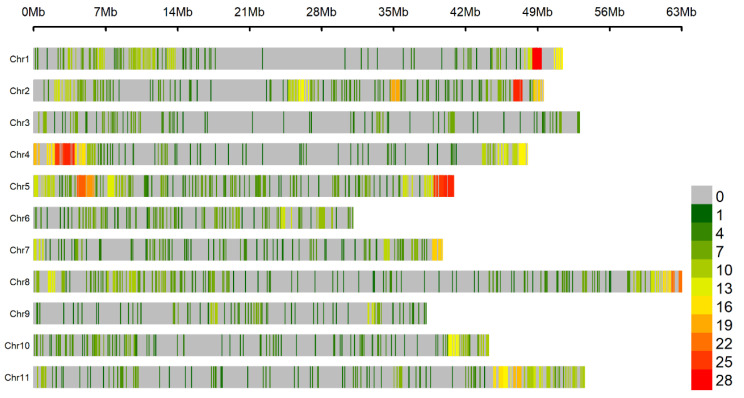
Density of 2001 SNPs in the MDP with 205 Mesoamerican genotypes. The different colors represent different density levels, and “Chr” refers to common bean chromosomes.

A total of 2001 SNPs were retained from the SNP calling, and these SNPs showed wide distribution over the 11 bean chromosomes (Figure 2). These markers were used for GWAS with the UFV01 and IAC18001 phenotypic data.

**Figure 3:**
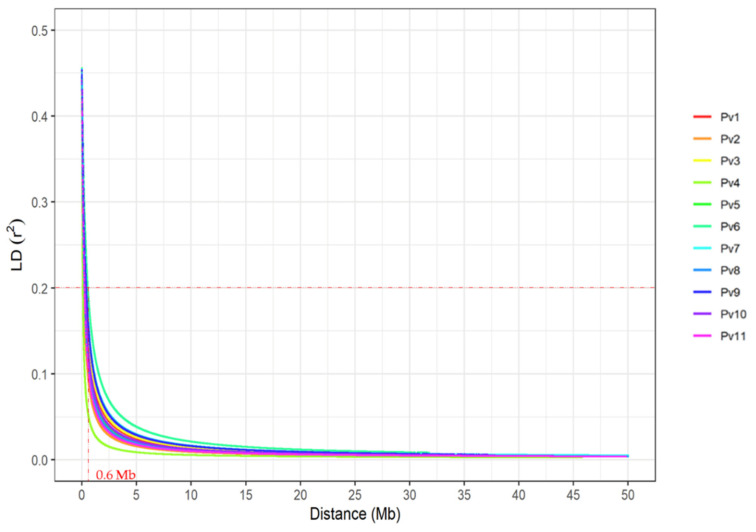
Linkage disequilibrium (LD) decay determined by the LD measurements (r^2^) based on 2001 filtered common beans against the distance between SNPs (Mb) for the 11 chromosomes (Pv) adjusted according to the model proposed by Hill and Weir [66] controlled for relatedness and structure in the MDP with 205 Mesoamerican genotypes.

Considering the small size of the bean genome (~597 Mb) and the mean of LD decay of r^2^ ≥ 0.2 was approximately 0.6 Mb for the Mesoamerican panel (Figure 3), the minimum number of SNPs required for good genome coverage and a satisfactory GWAS data was 1011, just over half of the number used in the present study (2001 SNPs).

**Figure 4:**
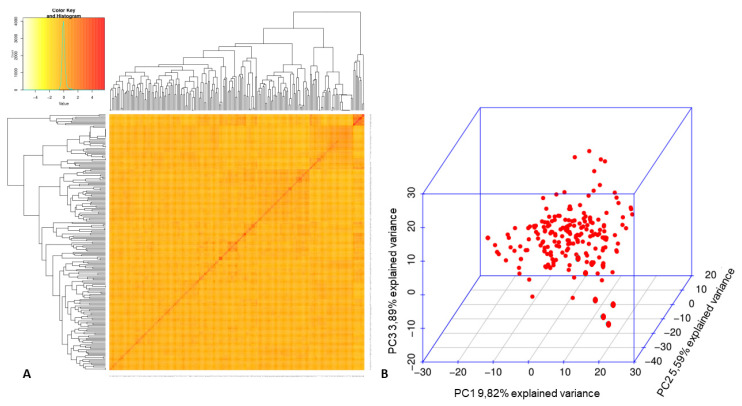
(**A**) Kinship plot of 205 common bean genotypes (MDP). (**B**) Principal component analysis calculated in the MDP with 205 genotypes and 2001 SNPs.

In association analysis, the kinship matrix is necessary as a covariate for correction of possible false-positive type associations (type I error) (Figure 4A), and the structuring matrix is necessary only in the presence of strong genetic structuring, which was not observed by principal component analysis (PCA), with a small value of the total variance in three dimensions (Figure 4B).

From the results obtained by PCA, the three principal components together explained only 19.3%, showing a small amount of the total variance explained by these components. Furthermore, no formation of sub-structuring was observed for the MDP, which may be explained by the Mesoamerican origin of the genotypes. Moreover, according to the BIC (Bayesian Information Criterion, Schwarz [60], zero was the best number of components to use in the association model, making it clear that there was no need to use principal components to correct type I error (i.e., false positives), avoiding overfit of the model (Table S2).

Despite the lower number of markers due to MAF (Minor Allele Frequency), heterozygosity, and missing data filters that would allow a greater number of associated SNPs, the GWAS results showed 11 significant SNPs, for the UFV01 and IAC18001 strains. The significant marker-phenotype association for the DSR and AUDPC parameters based on the measurement of symptoms of chlorosis, plant wilt, and vascular discoloration of the hypocotyl. These SNPs were at different genomic regions on chromosomes Pv01, Pv03, Pv04, Pv05, Pv07, Pv10, and Pv11, as shown by the resampling method and the Bonferroni test (α = 0.05). Of the eleven significant SNPs, six were within genes and five were close to candidate resistance genes, with distances ranging from 0.03 (Pv05) to 1.01 Mb (Pv01) from these genes. In accordance with haplotype blocks with LD markers, confidence intervals were defined for the annotation of candidate genes to identify direct and indirect associations through markers that may be in LD with the significant marker and the trait. The LD plot per chromosome considering r^2^ ≥ 0.2, and formation of haplotypic blocks, is highlighted with a color key (Figure S1).

**Figure 5:**
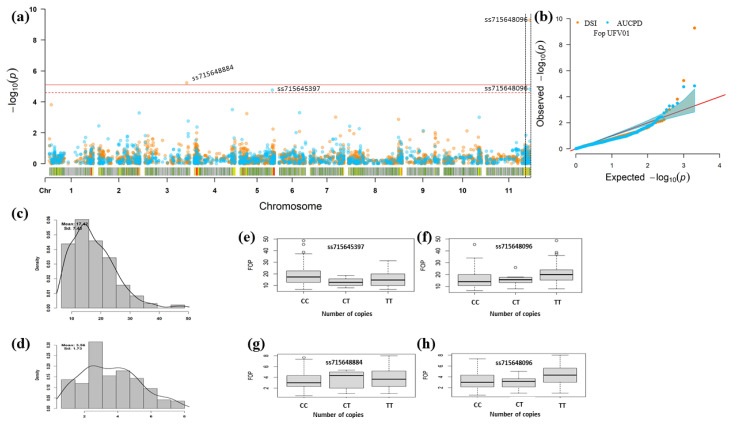
GWAS for *Fop* resistance in the MDP with 205 common bean genotypes with significant SNPs for the UFV01 strain using the DSR and AUDPC parameters and FarmCPU. (**a**) Manhattan plots and (**b**) Q-Q (Quantile-quantile) plots, with orange circles representing the *p*-values for DSR and blue circles the *p*-values for AUDPC. The dotted red line corresponds to the cut-offline obtained by the resampling method −log10(*p*) = 4.53 × 10^−5^, and the upper red line refers to the cut-offline obtained by the Bonferroni method (α = 0.05). (**c**,**d**) Histograms of the adjusted phenotypic means (BLUE) of AUDPC and DSR. (**e**–**h**) Boxplots of the relationship between the alleles and phenotype (*Fop* resistance) of each significant SNP for DSR and AUDPC.

The negative effect (−2.51) of the SNP associated with the UFV01 strain (SNP ss715645397 on Pv05) showed a decrease in the average values of *Fop* in the number of copies of the alternative allele “T” (Figure 5e). In contrast, the other significant SNPs had positive effects, with the highest value (2.08) for the SNP ss715648096 (Table 2), and the lowest value (0.37) for the SNP ss715646169 (Table 3). The marker ss715648096 (Pv11) was significant for the DSR and AUDPC parameters evaluated for the UFV01 strain and showed the phenotypic effect value of 0.73 for DSR and 2.08 for AUDPC; the two together explained 0.73% of the observed phenotypic variation (Table 2).

For evaluation of resistance to the IAC18001 *Fop* strain, seven SNPs located on chromosomes Pv01, Pv03, Pv04, Pv05, Pv07, and Pv10 showed high significance (Figure 5). The marker ss715646169 (Pv05) was associated with DSR and AUDPC evaluated for the IAC18001 strain. The ss715646169 marker showed the phenotypic effect value with 0.37 for DSR and 1.15 for AUDPC; the two together explained 0.18% of the observed phenotypic variation (Table 3).

The effect values tended to increase the *Fop* (UFV01) means in accessions having two copies of the alternative allele (“T”) compared to the reference allele (“C”) and to the heterozygote pattern (“C/T”) (Figure 5f–h).

**Figure 6:**
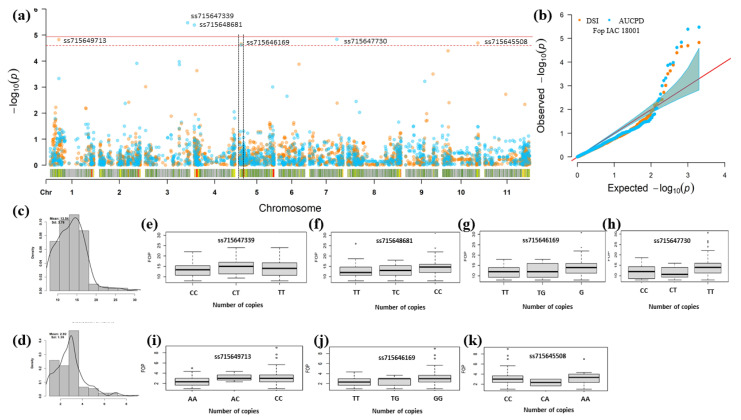
GWAS for *Fop* resistance in the MDP with 205 common bean genotypes with significant SNPs for the IAC18001 strain using the DSR and the AUDPC parameters and FarmCPU. (**a**) Manhattan plots and (**b**) Q-Q (Quantile-quantile) plots, with orange circles representing the *p*-values for DSR and blue circles the *p*-values for AUDPC. The dotted red line corresponds to the cut-offline obtained by the resampling method −log10(*p*) = 4.48 × 10^−5^, and the upper red line refers to the cut-offline obtained by the Bonferroni method (α = 0.05). (**c**,**d**) Histograms of adjusted phenotypic means (BLUE) of AUDPC and DSR. (**e**–**k**) Boxplots of the relationship between the allele and phenotype (*Fop* resistance) of each significant SNP for DSR and AUDPC.

Regarding the allelic effect profile, a similar pattern was observed for SNPs associated with the IAC18001 strain, in which all SNPs with two copies of the alternative allele showed a positive effect, tending to increase *Fop* averages in accessions (Figure 6e–k).

### 3.3. Genomic Regions Associated with Fusarium Wilt Resistance

Most of the significant SNPs showed different genomic regions when comparing the isolates, indicating that both strains characterized are from different races. Gene annotation identified a total of 78 genes associated with significant markers for the UFV01 strain (Table S3) and 329 genes for the IAC18001 strain (Table S4). In combined evaluation of the results obtained from the two strains, the SNPs located on Pv03 ss715647339 (*p*-value 3.36 × 10^−6^ IAC18001) and ss715648884 (*p*-value 5.81 × 10^−6^ UFV01) positioned at distance of 1.01 Mb associated with Fusarium wilt reaction, with candidate genes for both strains in this genomic region (Table S5).

An important cluster of 20 genes related to the transcription of proteins related to resistance mechanisms (R) such as the LRR- and NB-ARC domain-containing disease resistance protein is located next to the marker ss715648096 positioned at 51.50 Mb on Pv11. The cluster of 20 putative candidate genes was observed for the UFV01 strain considering DSR and AUDPC parameters at 0.03 Mb to 0.39 Mb from the significant marker in association analyses (Table 4).

The marker ss715646169 positioned at 1.99 Mb on Pv05 was significant for the IAC18001 strain (DSR and AUDPC parameters). This significant marker was positioned at 0.00 Mb and 0.56 from the candidate genes. The putative genes found were involved in disease resistance and related to important factors of transcription involved in biological signaling functions in drought tolerance and vascular diseases in plants (Table 5).

The significant markers ss715647339 (*p*-value 3.36 × 10^−6^ IAC18001) and ss715648884 (*p*-value 5.81 × 10^−6^ UFV01) were associated with *Fop* for both strains at 0.27 Mb and 0.51 from the candidate genes (Table 6). Among them, we found genes involved in root development mechanisms, in the transcription of disease-resistant proteins, and transcription factors involved in the important biological functions of signaling of drought tolerance and precursors of enzymes associated with flavonoid biosynthesis.

## 4. Discussion

The success of association mapping in identifying markers effectively associated with the trait depends on how well the population structure is corrected in the association model and on the existing levels of LD [76]. In a bean population, using a kinship matrix containing the population structure has been widely used in genome-wide association studies, successfully correcting the genetic relatedness between individuals using linear mixed models [48,77]. For association mapping in common bean, gene pools should be considered separately, because LD decays more rapidly within the Andean gene pool and is stronger within the Mesoamerican gene pool [5,78].

Regarding the Mesoamerican panel, the parameters observed in the current study agreed with those presented by [37], who evaluated a Mesoamerican carioca (cream-colored seed coat with brown stripes) panel, which is, in fact, part of the MDP used. The BIC test was performed for the first five components, and no PCs were required for any of the traits. The formation of haplotypic blocks within the LD markers ranging from 0.03 Mb (Pv05) to 1.01 Mb (Pv01) indicated that the markers evaluated represent the possible constituent haplotypes in the Mesoamerican panel [79].

*Fop* is genetically variable and often found in common bean growing in different countries and regions; up to now, seven pathogenic races related to geographical regions are cited in the literature [20,80,81], and new races like UFV01 and IAC18001 occur, supporting *Fop* pathogenic evolution [82]. However, mutations and recombination between avirulence genes (avr) in sexually reproducing pathogens are postulated as the mechanisms responsible for variation in races [83].

Resistance genes can be overcome by new or more virulent races; hence, broad-spectrum, durable resistance is needed [84]. In the current study, only 75 accessions (36.58%) showed resistance to both strains of *Fop*, demonstrating the difficulty of obtaining genotypes with resistance to different races of the fungus. Sala et al. (2006) evaluated 104 bean genotypes, of which 33% were resistant to the *Fop* 1, 2, 3, and 4 races, indicating the difficulty of finding cultivars with multiple resistance to the pathogen. Leitão et al. [25] evaluated a panel containing predominantly Andean accessions and the *Fop* race 06 and observed only 14 accessions (9.27%) with resistance to the fungus, with heritability values from 40.8% to 71.5% considering the DSR and AUDPC parameters (49% and 63%).

Important SNPs associated with QTL (Quantitative Trait *loci*) in the current study were associated with *Fop* resistance (represented by the parameters DSR and AUDPC) for the two strains tested (UFV01 and IAC18001). The differences reflect the varied resistance spectra exhibited by these accessions. Despite the experiments with both strains being conducted in few experiments under controlled conditions, some of the QTL identified in this study are confirmed by the literature, evidencing the robustness of results. However, the successful establishment of disease by the *Fop* pathogen demands a response in the plant defense system, and the entire molecular mechanism of pathogenesis remains to be elucidated to improve selective accuracy with additional experiments involving high-throughput phenotyping [85,86].

In bean, *Fop* penetrates the epidermis of the plant roots, invades the cortex, and colonizes the vascular tissue of the host plant, causing obstruction and wilting [14,15]. Pathogens other than *Fusarium* spp. can cause wilting in legumes; pathogens such as *Rhizoctonia* spp., *Verticillium* spp., and *Aphanomyces euteiches* [87]. Gupta et al. [88] confirmed that genes associated with the secondary cell wall are involved in the combined response of the plant to infection from wilt pathogens and to drought in *Arabidopsis thaliana.*

Furthermore, since we are likely dealing with polygenic inheritance with small additive genetic effects, increasing the sample size, thus maximizing the phenotypic diversity among the MDP, would enhance the power to recover meaningful associations [23,25]. Most of the SNPs associated by GWAS revealed that the genomic regions linked to *Fop* traits were located inside or near the candidate genes on Pv01, Pv03, Pv04, Pv05, Pv07, Pv10, and Pv11 (Table 2 and Table 3).

The Pv01 chromosome also showed a significant SNP, ss715649713, associated with DSR for the IAC18001 strain at the 1.01 Mb LD haplotype block, positioned within the Phvul.001G074800 (Appr-1-p processing enzyme family protein) gene. Appr-1-pase is an important and ubiquitous cellular processing [89]. Ubiquitination is a known mechanism in the regulation of plant defense against pathogens [90]. Recent evidence shows that ubiquitination plays a critical role in regulating plant responses to abiotic stresses and plant tolerance of adverse environmental conditions [91]. The ubiquitination mechanism may also be associated with actions on specific components for stress signaling [92].

On Pv03, two significant SNPs associated with the *Fop* reaction were found, the ss715647339 (IAC18001) and ss715648884 (UFV01) positioned at a distance of 1.01 Mb, and showed potential candidate genes involved in root development mechanisms (Phvul.003G258100) and in presumed disease-resistance proteins (Phvul.003G258700, Phvul.003G258800, and Phvul.003G260300). The Phvul.003G258400 gene is associated with the putative Cytochrome P450 superfamily protein also in this region family members can act in the control of abscisic acid (ABA) production that are involved in critical processes in plant growth and development. They can also act in biotic and abiotic stress responses [93,94] and the formation of secondary metabolites, such as terpenoids, flavonoids, steroids, alkaloids, phenylpropanoids, glucosinolate, and cyanogenic glycoside all of which are typically made as part of host defense [95].

The SNP ss715648681 identified on Pv04 associated with AUDPC for the IAC18001 strain is positioned within the Phvul.004G001900 gene (MATE efflux family protein). In plants, MATE transporters have been directly or indirectly implicated in mechanisms of disease resistance [96], in the transport of diverse types of secondary metabolites, such as alkaloids [97], flavonoids [98,99], anthocyanidins [100], and hormones, such as salicylic acid (SA) and ABA, and in drought tolerance [101]. Mandal et al. [102] demonstrated that the induced resistance observed in tomato against *Fusarium oxysporum* f. sp. *lycopersici* (Fol) might be a case of salicylic acid-dependent systemic acquired resistance.

Another significant SNP, ss715645397, was found in Pv05 associated with AUDPC for UFV01 at 0.004 Mb from the Phvul.005G152600 gene (ARM repeat superfamily protein). The Armadillo (ARM) domain has motifs with the structure of repeat proteins, such as Leucine-rich repeats (LRR), that have been extensively studied in plants, suggesting a critical role of these repeating peptides in plant cell physiology, plant stress, and plant development [103]. In this region close to the marker, Nakedde et al. [46] identified a QTL mapped in a recombinant inbred line (RIL) population that accounted for 9.20% to 10.06% of phenotypic variation associated with Fusarium Root Rot (FRR) and root architecture traits. This QTL was located at 39.22 Mb in a 0.31 Mb interval on Pv05.

Another candidate gene associated with the ss715646169 marker positioned at 1.99 Mb on Pv05 (between 0.0 Mb and 0.56 Mb) for DSR and AUDPC of the IAC18001 strain. This marker was positioned within the Phvul.005G022100 gene (Cellulose synthase family protein). The cellulose synthase (CesA) superfamily genes are among the most important agents involved in the biosynthesis of plant cell walls, which are mainly composed of biopolymers such as celluloses, hemicelluloses, pectins, and lignins [104]. Among the several defense mechanisms in the plant–pathogen resistance interaction, structural changes must be highlighted. These structural changes lead to strengthening of the plant cell wall by the deposition of callose, followed by lignification, a phenomenon that can be determinant in a resistance or susceptibility reaction in interaction with *Fusarium oxysporum*, with the possibility of quantitative differences in response [105].

Our results showed a group of candidate gene associated with the ss715646169 marker are the genes related to the zinc finger domain (Phvul.005G016200; Phvul.005G019900; Phvul.005G020000 and Phvul.005G022000). Zinc finger proteins play a crucial role in many metabolic pathways, as well as in stress response and defense in plant-pathogen interactions to the defense of plants, and may be associated with a JA-dependent defense pathway [106,107]. The SNP ss715647730 identified on Pv07 and associated with AUDPC for IAC18001 was positioned at 0.01 Mb from the Phvul.007G199600 gene (drought-responsive family protein). Although drought-responsive proteins exhibit various patterns depending on plant species, genotypes, and stress intensity, proteomic analyses show that dominant changes occurred in sensing and signal transduction, reactive oxygen species scavenging, osmotic regulation, gene expression, protein synthesis/turnover, cell structure modulation, and carbohydrate and energy metabolism [108].

Leitão et al. [25] performed association mapping for *Fop* race 06 using a panel of 133 common bean accessions from Portugal and observed significant associations detected for DSR and AUDPC on the Pv04, Pv05, Pv07, and Pv08 chromosomes. They noted that the DART03480 marker on Pv04 was at a small distance of approximately 0.1 Mb from the ss715648681 marker, which was also detected in our study.

The Pv10 chromosome showed a significant SNP, ss715645508, positioned at a distance of 0.001 Mb from the Phvul.010G137000 gene (SNARE-like superfamily protein). This gene may be considered a novel determinant of salinity/drought tolerance and a potential candidate to increase salinity and drought tolerance in crop plants [109]. Erfatpour et al. [110] identified a QTL in this same genomic region between 39.97 Mb and 40.29 Mb, with forty candidate genes associated with non-darkening (ND) in seed coat color at 1.6 Mb from the significant marker in our study.

Linkage mapping reported genomic regions associated with *Fop* resistance to race 04 [23]. The authors identified significant markers positioned on Pv03, Pv10, and Pv11, and a QTL of greater effect that explained 63.5% of the phenotypic variance on Pv10. A SCAR marker (U20.750) linked to this QTL was developed, with evaluation in Andean and Mesoamerican germplasm, and the marker had high accuracy in Mesoamerican accessions [111].

Gene annotation allowed the identification of candidate genes associated with putative effects in disease-resistance mechanisms (R), such as a cluster of 20 candidate genes annotated as “leucine-rich repeat-containing protein” (LRR), with distances from 0.03 Mb from the Phvul.011G200300 gene up to 0.39 Mb from the Phvul.011G203100 gene positioned close to the ss715648096 marker on Pv11 associated with DSR and AUDPC for UFV01 (Table 4). The region of 51.50 Mb associated with the significant ss715648096 marker on Pv11 corroborates previous studies, and the region being associated with other important fungal diseases of common bean, such as anthracnose, by the association of marker S11_51790295 to race 73 of *Colletotrichum lindemuthianum* (the anthracnose pathogen), positioned at a distance of approximately 0.20 Mb [112]. The identification of LRR receptor-like protein kinases (PK) and their role in adaptive selection supports prior literature indicating a co-evolution of common bean and the anthracnose fungus [44,113].

The GWAS of the Mesoamerican panel also revealed the S11_50585184 marker at 0.91 Mb from the ss715648096 marker associated with *Fop* that is related to the Phvul.011G192400 (NBS-LRR with typical NB-ARC domain) gene associated with *Rhizoctonia solani* resistance on Pv11 [36]. The response to different soil diseases may be because the NB-ARC domain contains a functional ATPase region that regulates the resistance, and this domain interacts with the nucleotide-binding domain in order to exchange the nucleotides that are associated with activating ATPase change, which, in turn, reshapes to NB-ARC ATPase and alters resistance specificity and the possibility that the LRR interacts with similar elicitors from both pathogens [114,115].

Hoyos-Villegas et al. [116] used the GWAS procedure for wilting score associated with drought-tolerant genotypes and reported one significant association at the SNP ss715639678, which is located at the end of Pv11, in a region that was found to be in high LD, with 1131 genes. In addition, gene ontology enrichment analysis revealed 19 biological processes and 30 molecular functions that were significantly associated. Myers et al. [117], using GWAS for finding markers associated with total phenolic content (TPC), identified 11 QTNs linked with TPC, especially the SNP ss715650328 at 52.96 Mb on Pv11. Various biological functions may be related, including the production of compounds such as phenolic acids, flavonoids, and proanthocyanidins, which are the main polyphenols associated with plant defense and postharvest darkening in common bean [118,119].

The physical barriers that act at different levels in defending plants inhibit the penetration and colonization of plant tissues by the pathogen, associated with biochemical reactions in the host cells that produce toxic substances and/or create adverse conditions for growth of the pathogen inside the plant. Therefore, substances produced in the host cells, before or after infection, contribute significantly to resistance [120].

Some signaling components, such as phytohormones, combined with functional gene transcription factors and their regulators, are involved in responses to combined abiotic and biotic stresses in plants, factors that can be modulated according to environmental conditions [121]. The effect of water can modulate the response of the plant to pathogens, in which several pathogens translocate virulence proteins (effectors) into host cells to target different components of the plant [122].

Chen et al. [27], using whole transcriptome and metabolome, showed bean-*Fop* pathosystem includes different and effective defense pathways comprising of a complex resistance network of structural, signaling, and chemical responses. The authors demonstrated the validation of differentially expressed genes located in Pv03, Pv04, Pv07, Pv08 and Pv11 by qRT-PC showing strong roles in signaling routes such as salicylic acid (SA), jasmonate, and ethylene. *Fop* also induced the flavonoid biosynthesis pathway which was the most significantly enriched one in response to *Fop*’s infection.

Xue et al. [123] using the cDNA amplified fragment length polymorphisms (cDNA-AFLPs), found five transcript-derived fragments involved in the mechanism of plant hormone regulation. These five genes belonged to the jasmonate, auxin, Abscisic acid (ABA), and SA-dependent pathways can be implicated to play a role in the plant’s defense responses.

After exposure to the pathogen, the plant starts a signaling network mediated by protein kinases, such as mitogen-activated protein kinases (MAPK) and begins a process of recognition of pathogen-associated molecular patterns (PAMPs) through their PAMP-recognition receptors (PRRs), known as pattern-triggered immunity (PTI) and pathogen effector-triggered immunity (ETI), two important mechanisms for averting disease attacks [124].

## 5. Conclusions

In our study, the SNPs and putative candidate genes associated with *Fop* resistance may help to broaden understanding of the pathways involved in bean response to *Fop* infection. Significant markers related to *Fop* resistance showed common response mechanisms similar to other bean diseases, in association with root architecture traits, which is indeed the entrance of *Fop* infection. These genes thus affect the drought-tolerance response of the plants and the production of phenolic compounds, indicating a complex gene network with pleiotropic effects in common beans related to this disease. However, we recommend future studies involving field conditions using high-throughput phenotyping and different approaches (i.e., linkage mapping, transcriptome and metabolome) to validate the results obtained with Mesoamerican derived beans. The putative candidate genes associated with the SNPs in the current study increase the number of functional markers available to facilitate possible application to breeding by genomic selection for *Fop* resistance in common bean.

## Figures and Tables

**Table 1:** Broad-sense heritability, selective accuracy for resistance, overall mean of controls for two *Fop* strains evaluated for the Mesoamerican Diversity Panel (MDP) and likelihood radio test (LRT) of random effects of the DSR and AUDPC.

Source of Variation	UFV01 Strain		IAC 18001 Strain	
MDP	DSR	AUDPC	DSR	AUDPC
Genotypes	1220.14 **	3075.29 **	1024.71 **	2367.56 **
Broad-sense heritability	0.63 ± 0.09	0.57 ± 0.09	0.55 ± 0.08	0.49 ± 0.08
Selective accuracy	0.91	0.89	0.88	0.86
Mean resistant control ^1^	1.67 ± 0.20	10.00 ± 0.81	1.66 ± 0.11	9.33 ± 0.63
Mean susceptible control ^2^	7.67 ± 0.56	28.00 ± 2.12	7.23 ± 0.86	24.60 ± 2.17
Mean	3.60 ± 1.73	17.40 ± 7.48	2.96 ± 1.35	13.60 ± 3.78
Pearson Correlation ^3^	0.87		0.86	

** *p* < 0.01 by the LRT and analysis of deviance; ^1^ IAC Milênio cultivar, ^2^ BRS Estilo cultivar.^3^ Pearson Correlation among DSR and AUDPC for each strain.

**Table 2:** SNPs detected for the DSR and AUDPC for the UFV01 *Fop* strain for 205 common bean genotypes and 2001 SNPs: SNP’s positions in mega base pairs (Mb).

Trait	Chr ^1^	Position v2.1 ^2^	SNP	*p*-Value	MAF ^3^	Effect ^4^	Alleles	R^2^% ^5^
DSR	Pv03	49,467,577	ss715648884	5.81 × 10^−6^	0.21	0.63	C ^e^/T ^f^	0.16
	Pv11	51,500,684	ss715648096	5.27 × 10^−10^	0.32	0.73	C ^e^/T ^f^	0.64
AUDPC	Pv05	38,267,303	ss715645397	1.73 × 10^−5^	0.17	−2.51	C ^e^/T ^f^	0.09
	Pv11	51,500,684	ss715648096	2.59 × 10^−5^	0.32	2.08	C ^e^/T ^f^	0.09

^1^*P. vulgaris* chromosome; ^2^ Position in base pairs (bp); ^3^ Minor allele frequency; ^4^ A positive effect of the allelic variant represents an increase in susceptibility, while a negative effect represents an increase in resistance to Fusarium wilt; ^5^ Variance explained by each SNP-trait association (%); ^e^ Allelic reference; ^f^ Allelic variant.

**Table 3:** SNPs detected for the DSR and AUDPC for the IAC18001 *Fop* strain for 205 common bean genotypes and 2001 SNPs: SNP’s positions in mega base pairs (Mb).

Trait	Chr ^1^	Position v2.1 ^2^	SNP	*p*-Value	MAF ^3^	Effect ^4^	Alleles	R^2^% ^5^
DSR	Pv01	10,289,227	ss715649713	1.50 × 10^−5^	0.18	0.45	A ^e^/C ^f^	0.09
	Pv05	1,990,853	ss715646169	2.20 × 10^−5^	0.22	0.37	T ^e^/G ^f^	0.09
	Pv10	41,966,104	ss715645508	3.02 × 10^−5^	0.06	0.64	C ^e^/A ^f^	0.09
AUDPC	Pv03	50,473,206	ss715647339	3.36 × 10^−6^	0.46	1.05	C ^e^/T ^f^	0.16
	Pv04	155,465	ss715648681	4.13 × 10^−6^	0.50	0.98	T ^e^/C ^f^	0.09
	Pv05	1,990,853	ss715646169	2.42 × 10^−5^	0.22	1.15	T ^e^/G ^f^	0.09
	Pv07	32,298,702	ss715647730	3.02 × 10^−5^	0.19	1.21	C ^e^/T ^f^	0.09

^1^*P. vulgaris* chromosome; ^2^ Position in base pairs (bp); ^3^ Minor allele frequency; ^4^ A positive effect of the allelic variant represents an increase in susceptibility, while a negative effect represents an increase in resistance to Fusarium wilt; ^5^ Variance explained by each SNP-trait association (%); ^e^ Allelic reference; ^f^ Allelic variant.

**Table 4:** Annotation of candidate genes with a confidence interval (0.464 Mb) of each significant SNP ss715648096 associated with the Fusarium wilt strain UFV01 for DSR (*p*-value 5.27 × 10^−10)^ and AUDPC (*p*-value 2.59 × 10^−5^) for 205 common bean genotypes and 2001 SNPs: SNP’s positions in mega base pairs (Mb).

Gene	Distance ^1^	Description
Phvul.011G200300	0.035	NB-ARC domain-containing disease resistance protein
Phvul.011G200800	0.069	LRR/NB-ARC domain-containing disease resistance protein
Phvul.011G200820	0.103	NB-ARC domain-containing disease resistance protein
Phvul.011G200840	0.109	LRR and NB-ARC domain-containing disease resistance protein
Phvul.011G200860	0.111	NB-ARC domain-containing disease resistance protein
Phvul.011G200880	0.122	LRR and NB-ARC domain-containing disease resistance protein
Phvul.011G200900	0.141	NB-ARC domain-containing disease resistance protein
Phvul.011G201000	0.151	LRR and NB-ARC domain-containing disease resistance protein
Phvul.011G201101	0.159	NB-ARC domain-containing disease resistance protein
Phvul.011G202000	0.248	NB-ARC domain-containing disease resistance protein
Phvul.011G202200	0.264	LRR and NB-ARC domain-containing disease resistance protein
Phvul.011G202366	0.295	NB-ARC domain-containing disease resistance protein
Phvul.011G202432	0.297	NB-ARC domain-containing disease resistance protein
Phvul.011G202601	0.341	LRR and NB-ARC domain-containing disease resistance protein
Phvul.011G202750	0.343	LRR and NB-ARC domain-containing disease resistance protein
Phvul.011G202800	0.344	Disease resistance protein (TIR-NBS-LRR class) family
Phvul.011G202900	0.347	LRR and NB-ARC domain-containing disease resistance protein
Phvul.011G202966	0.365	NB-ARC domain-containing disease resistance protein
Phvul.011G203032	0.388	LRR and NB-ARC domain-containing disease resistance protein
Phvul.011G203100	0.393	LRR and NB-ARC domain-containing disease resistance protein

^1^ Distance among de significant SNP and candidate gene (Mb).

**Table 5:** Annotation of candidate genes with a confidence interval (0.694 Mb) of each significant SNP ss715646169 associated with the Fusarium wilt (*Fop*) strain IAC18001 for DSR (*p*-value 2.20 × 10^−5^) and AUDPC (*p*-value 2.42 × 10^−5^) for 205 common bean genotypes and 2001 SNPs: SNP’s positions in mega base pairs (Mb).

Gene	Distance ^1^	Description
Phvul.005G016200	0.561	Zinc ion binding
Phvul.005G016300	0.556	Late embryogenesis abundant protein, group 1 protein
Phvul.005G016500	0.541	Disease resistance protein (TIR-NBS-LRR class), putative
Phvul.005G017000	0.510	Protein kinase superfamily protein
Phvul.005G018300	0.415	Proline-rich family protein
Phvul.005G019900	0.261	Zinc finger (CCCH-type/C3HC4-type RING finger) protein
Phvul.005G020000	0.251	Zinc finger (CCCH-type/C3HC4-type RING finger) protein
Phvul.005G020100	0.247	Ubiquitin carboxyl-terminal hydrolase family protein
Phvul.005G020600	0.197	Putative methyltransferase family protein
Phvul.005G021300	0.129	Microtubule-associated proteins
Phvul.005G022000	0.018	CCCH-type zinc finger protein with ARM repeat domain
Phvul.005G022100	0.000	Cellulose synthase family protein

^1^ Distance among de significant SNP and candidate gene (Mb).

**Table 6:** Annotation of candidate genes with a confidence interval (1.001 Mb) of significant SNPs ss715647339 (*p*-value 3.36 × 10^−6^ IAC18001) and ss715648884 (*p*-value 5.81 × 10^−6^ UFV01) associated with Fusarium wilt for 205 common bean genotypes and 2001 SNPs: SNP positions in mega base pairs (Mb).

Gene	Distance ^1^	Description
Phvul.003G258100	0.277	Lateral root primordium (LRP) protein-related
Phvul.003G258400	0.348	Cytochrome P450 superfamily protein
Phvul.003G258700	0.381	Leucine-rich repeat (LRR) family protein
Phvul.003G258800	0.386	Leucine-rich receptor-like protein kinase family protein
Phvul.003G259700	0.447	Serine carboxypeptidase S28 family protein
Phvul.003G260000	0.465	Serine carboxypeptidase S28 family protein
Phvul.003G260100	0.481	B-box type zinc finger family protein
Phvul.003G260200	0.495	ATP binding microtubule motor family protein
Phvul.003G260300	0.514	Leucine-rich receptor-like protein kinase family protein

^1^ Distance among de significant SNP and candidate gene (Mb).

## Data Availability

Not applicable.

## Supplementary Materials

The following are available online at https://www.mdpi.com/article/10.3390/genes12050765/s1, Figure S1: LD plot considering r^2^ ≥ 0.2 for LD haplotype on chromosomes Pv01, Pv03, Pv04, Pv05, Pv07, Pv10, and Pv11, Table S1: Summary of phenotypic and genotypic data for bean lines within the Mesoamerican Diversity Panel (MDP), Table S2: Bayesian Information Criterion (BIC) test; larger is better, according to Schwarz, (1978), Table S3: Gene annotation for the significant SNPs from GWAS in MDP genotypes for the UFV01 strain (https://phytozome.jgi.doe.gov/pz/portal.html, accessed on 14 May 2021. Table S4: Gene annotation for the significant SNPs from GWAS in MDP genotypes for the *Fop* IAC18001 strain (https://phytozome.jgi.doe.gov/pz/portal.html, accessed on 14 May 2021. Table S5: Gene annotation for the common significant SNPs from GWAS in MDP genotypes for *Fop* UFV01 and IAC18001 strains (https://phytozome.jgi.doe.gov/pz/portal.html, accessed on 14 May 2021.
