# Supplementary figures and images for: Genome-Wide Association Study Reveals Genomic Regions Associated with Fusarium Wilt Resistance in Common Bean
Source: Genes (Basel). 2021 May 18;12(5):765. doi: 10.3390/genes12050765 (PMC8157364; doi:10.3390/genes12050765)

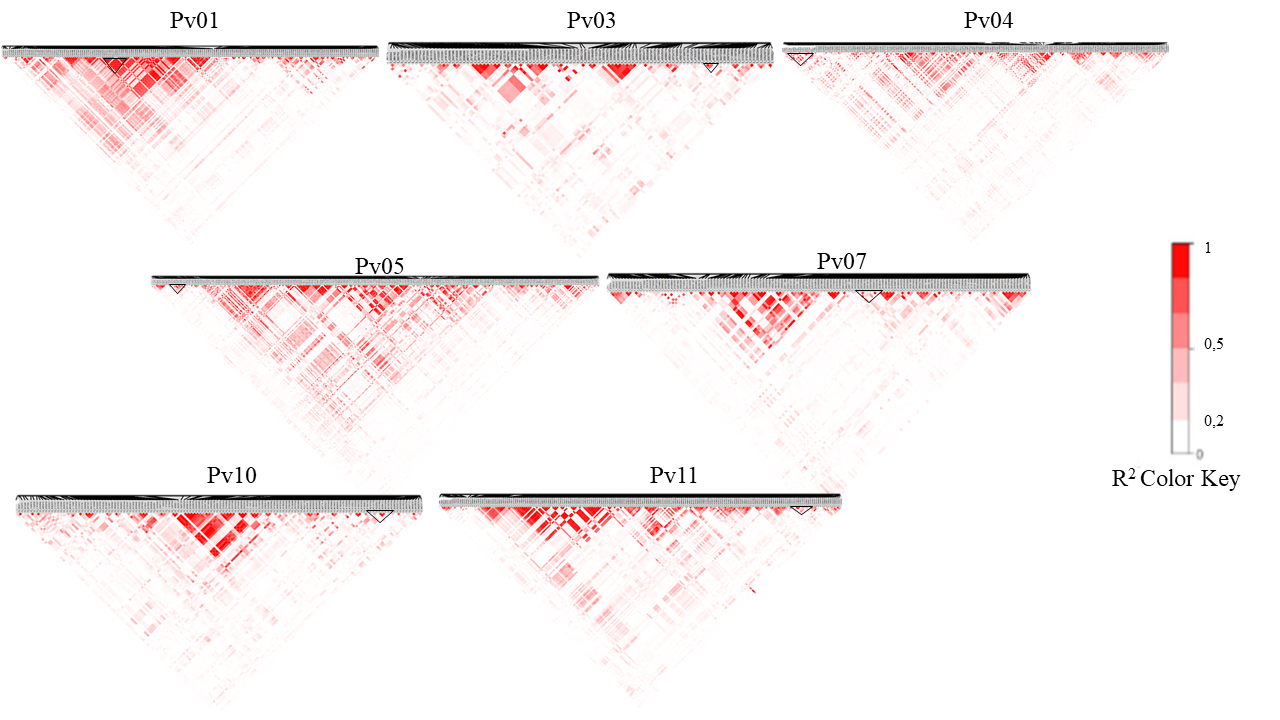

Supplement: Supplementary file 1 [file genes-12-00765-s001.zip › Supplementary/Figure S1 LD.tif]
